# Iron requirements and uptake strategies of the globally abundant marine ammonia-oxidising archaeon, *Nitrosopumilus maritimus* SCM1

**DOI:** 10.1038/s41396-019-0434-8

**Published:** 2019-05-10

**Authors:** Roxana T. Shafiee, Joseph T. Snow, Qiong Zhang, Rosalind E. M. Rickaby

**Affiliations:** Department of Earth Sciences, University of Oxford, South Parks Road, Oxfordshire, OX1 3AN UK

**Keywords:** Biogeochemistry, Archaeal physiology, Biogeochemistry

## Abstract

Ammonia-oxidising archaea (AOA) mediate the rate-limiting step of nitrification, the central component of the marine nitrogen cycle that converts ammonia to nitrite then nitrate. Competition with phytoplankton for ammonium and light inhibition are considered to restrict AOA activity to below the photic zone, but observations of surface nitrification now demand a further understanding of the factors driving AOA distribution and activity. Pico- to nanomolar concentrations of iron (Fe) limit the growth of microorganisms in a significant portion of the world’s surface oceans, yet there is no examination of the role of Fe in AOA growth despite the process of ammonia oxidation being considered to rely on the micronutrient. Here we investigate the Fe requirements and Fe uptake strategies of the *Nitrosopumilus maritimus* strain SCM1, a strain representative of globally abundant marine AOA. Using trace metal clean culturing techniques, we found that *N. maritimus* growth is determined by Fe availability, displaying a free inorganic Fe (Fe′) half saturation constant 1–2 orders of magnitude greater for cell growth than numerous marine phytoplankton and heterotrophic bacterial species driven by a reduced affinity for Fe′. In addition, we discovered that whilst unable to produce siderophores to enhance access to Fe, *N. maritimus* is able to use the exogenous siderophore desferrioxamine B (DFB), likely through a reductive uptake pathway analogous to that demonstrated in phytoplankton. Our work suggests AOA growth in surface waters may be Fe limited and advances our understanding of AOA physiology on the cellular and mechanistic levels with implications for ecosystem dynamics and the biogeochemical N-cycle.

## Introduction

Ammonia oxidation is the first and rate-limiting step of nitrification, mediated by ammonia-oxidising bacteria and archaea (AOB and AOA). Nitrification links the reduced and oxidised components of the nitrogen (N) cycle through the conversion of ammonium (NH_4_^+^) to nitrite (NO_2_^−^) and then nitrate (NO_3_^−^). Greater ratios of NO_3_ to NH_4_ in surface waters are shown to promote the growth of faster-sinking microphytoplankton (such as diatoms) relative to picophytoplankton, thereby driving increased carbon export to the deep ocean [1–5]. Nitrification rates also have a climatic impact through the release of the by-product nitrous oxide (N_2_O), a potent greenhouse gas [6, 7]. In the oceans AOA, belonging to the phylum *Thaumarchaeota*, largely outnumber their bacterial counterparts [8, 9] and are among the most ubiquitous and abundant microbes, constituting ~20% of total marine prokaryotic cells [10]. Due to their key role in N-biogeochemical cycling, investigation has focused on understanding the factors that determine the distribution of *Thaumarchaeota* and nitrification rates throughout the water column. Photoinhibition of AOA [11–14] and competition with phytoplankton for NH_4_^+^ [15–17] have both been evoked as explanations of why peak nitrification rates typically occur at the base of the euphotic zone. However, observations of high nitrification rates in the euphotic zone indicate that this is not universally the case [18], suggesting that our understanding of the factors shaping the distribution of AOA is incomplete. No studies to date have directly examined the role of the trace metal iron (Fe) in *Thaumarchaeota* growth, despite a vast body of evidence showing that Fe influences the growth of a range of marine N-cycling microorganisms in a significant portion of the world’s oceans [19, 20, 21]. The only study to date to directly examine the role of trace metals in *Thaumarchaeota* growth, demonstrated that the marine model species *Nitrosopumilus maritimus* (strain SCM1) has a high copper requirement, driven by numerous multi-Cu oxidases and blue Cu-proteins resembling sulpho- and plastocyanin enzymes [22, 23]. In contrast, AOB utilise a greater number of Fe-based enzymes and fewer Cu-proteins [24] suggesting that AOB may possess a lower Cu requirement. Whether oceanic metal availability shapes the ecological niche separation of AOB vs. AOA, in a similar way to ammonium [25, 26] in the oligotrophic oceans has yet to be explored. In addition, it remains unknown which Fe-substrates are available for uptake by *N. maritimus*, the strategies *N. maritimus* employs to gain access to low levels of Fe in the oceans, and how together these phenotypic traits may influence the distribution of AOA throughout the water column. In order to aid in Fe acquisition, some marine microorganisms produce high affinity iron(III) binding ligands called siderophores, which acts to increase the bioavailability of Fe to some microorganisms [27]. The ability of microbes to utilise different classes of siderophores, taken up either by siderophore specific membrane channels or by reduction of Fe-chelates [28–30], can provide microorganisms with a competitive advantage in iron-poor niches of the ocean.

Here we use trace metal clean culture techniques to examine the role of Fe in the growth of *N. maritimus* strain SCM1 with the aim of understanding how Fe in the oceans may drive the distribution of marine AOA species. We specifically probe the role of environmentally important Fe substrates in  AOA growth including unchelated inorganic Fe (Fe′) and organic siderophore bound Fe. Our study is the first to shed light on the role of Fe in AOA growth, presenting implications for how Fe in the marine environment may shape the ecological niche that AOA occupy in the water column.

## Materials and methods

### Archaeal cultures and growth media

Polycarbonate culture vessels and culturing apparatus were acid cleaned (10% HCl v/v, 24 h) and UV-sterilised prior to use. Triplicate cultures of *Nitrosopumilus maritimus* strain SCM1 (herein referred to as SCM1) were grown in SCM medium [31] and maintained at 28 °C in the dark. Macronutrients used in media preparation were treated with Chelex-100 resin (BioRad, Watford, UK) to remove trace metal contaminants [32]. The final pH of the media was 7.5. To investigate the effect of unchelated inorganic Fe concentration ([Fe′]) on SCM1 growth, ethylenediamine tetraacetic acid (EDTA; Merck, Darmstadt, Germany) was used to buffer Fe and other metals in cultures; [Fe′] was controlled by varying the addition of FeCl_3_ and maintaining a constant concentration of EDTA at 12 µmol L^−1^. We did not observe difference in growth rate in cultures with equivalent [Fe′] (7500 pmol L^−1^) but with varying [EDTA]; cultures with 12 µmol L^−1^ EDTA, relative to regular SCM media with 7.5 µmol L^−1^ [EDTA], did not produce an inhibitory effect (data not shown). [Fe′] in cultures was calculated using Visual Minteq software [33], including background Fe contamination of 50 nmol L^−1^ from basal SCM medium with included macronutrients and vitamins but without added trace metals, determined using inductively coupled plasma mass spectrometry [34]. Cultures were acclimated to metal concentrations stated by transferring cultures consecutively into new media until growth rates did not vary with statistical significance (ANOVA, *p* < 0.05).

Growth was determined by measuring concentrations of nitrite ([NO_2_^−^]) spectrophotometrically [35], which was observed to correlate with cell counts at both high and low [Fe′] (Supplementary Fig. 1). Specific growth rate (d^−1^) was calculated over the linear phase of semi-log plots of nitrite concentration over time. SCM1 and published phytoplankton and marine heterotrophic bacterial growth rate data [36–43] in response to [Fe′] were fit to a Monod function:

1 $$\mu = \frac{{\mu _{{\mathrm{max}}} \cdot [{\mathrm{Fe}}\prime ]}}{{K_s + \left[ {{\mathrm{Fe}}\prime } \right]}}$$

Whereby *µ* is growth rate (d^−1^), *µ*_max_ is maximum growth rate, [Fe′] is the concentration of unchelated inorganic Fe and *K*_*s*_ is the growth half saturation constant.

### Calculation of steady state uptake rate constant

In order to compare the Fe-uptake rates of SCM1 with published uptake rates of other marine microorganisms compiled in our study ([28, 30, 37, 44–49]; Supplementary Table 1), we calculated the steady state uptake rate (*ρ*_ss_) in mol Fe per cell h^−1^ and steady state uptake constant (*K*_in_) in L cell^−1^ h^−1^ as defined in [29]. Cell numbers used in our calculations were obtained by using a conversion factor of 6.01 × 10^10^ cells per mol NO_2_^−^ derived from measurements of cell counts with increasing [NO_2_^−^] (Supplementary Fig. 1).

2 $$\rho _{ss} = Q \cdot \mu$$

3 $$K_{in} = \frac{{\rho _{ss}}}{{[S]}}$$

Whereby *Q* is equal to the cellular Fe quota (mol Fe cell^−1^), *µ* is specific growth rate (h^−1^) and *S* is the concentration of Fe substrate (either Fe′ or FeDFB) (mol L^−1^). Furthermore, we were able to normalise the uptake rate constant to cell surface area (*K*_in_ /S.A.) in order to obtain an uptake constant independent of cell surface area and therefore proportional to the affinity of the uptake system. SCM1 surface area was calculated based on average dimensions of cells imaged using scanning electron microscopy (Supplementary Methods). SCM1 cells were rod-shaped with a diameter of 0.1–0.2 µm and length of 0.45–0.58 µm (Supplementary Fig. 2); cell surface area was calculated using an average diameter of 0.15 µm and length of 0.52 µm. These dimensions agree with the previous analyses of SCM1 size [31], which showed rod-shaped cells with a diameter of 0.17–22 µm and length of 0.5–9 µm. All steady state uptake constants used in our comparison were compiled and calculated by Lis et al. [30] from published datasets (Supplementary Table 2).

### Siderophore production and Fe-complex uptake pathway

To examine whether SCM1 is able to grow using siderophore bound Fe, SCM1 was cultured in medium with the addition of desferrioxamine B mesylate (DFB; Merck), a fungal siderophore that has a greater stability constant (logK´_FeL,Fe′_ = 11.8) for Fe(III) compared with EDTA (logK´_FeL,Fe′_ = 8.6, pH 8) [50, 51]. [Fe′] in DFB buffered media was calculated using the equation [Fe′] = [FeDFB]/([DFB´] × [K_Fe′DFB_^cond^]). A 1.3-fold excess of DFB was added over Fe, resulting in [Fe′] of 5.2 × 10^−9^ pmol L^−1^, considered to be negligible, thereby making Fe bound to DFB (FeDFB) the main Fe substrate in these experiments. EDTA concentrations were kept constant at 12 µmol L^−1^ EDTA to buffer other trace metals as done in previous studies [46, 47].

Siderophore secretion by SCM1 was tested using a liquid Chrome Azurol S (CAS) assay [52]. Prior to this assay, potential siderophores were preconcentrated from the supernatant of 300 and 1050 pmol L^−1^ Fe′ cultures (grown in the absence of EDTA), using Solid Phase Extraction (SPE) cartridges (Isolute ENV+, 1 g 25 mL, Biotage, Hengoed, UK). Cartridges were precleaned with 5 ml methanol and 5 ml 11.2 mmol L^−1^ ammonium carbonate (pH 7.5). One litre of 0.1 µm-filtered culture supernatant was passed through the column at a flow rate of 8 ml min^−1^, followed by 5 ml of 11.2 mmol L^−1^ ammonium carbonate to remove salts. Siderophores were eluted with 5 ml of 1:20:80 (v/v/v) formic acid/water/methanol. The SPE eluent from all samples was dried under high purity N_2_ to 100 μL and then made up to 500 μL with 0.1 % (v/v) formic acid (Sigma-Aldrich) prior to analysis. The sensitivity of this method was tested by preconcentrating 1 L of 1–100 pmol L^−1^ DFB solutions, which are typically undetectable by the CAS Assay, using the SPE columns. The lowest concentration detected using this method was 10 pmol L^−1^ DFB, which was used as a positive control. Uninoculated EDTA-free SCM media passed through the SPE column was used as a blank. Spectra were measured between 400 and 800 nm and siderophore production was observed as a decrease in the absorbance peak at 630 nm.

We used the ferrozine assay [29] to identify whether SCM1 adopts a reductive Fe-uptake pathway in Fe acquisition. SCM1 cultures acclimated to total [Fe] of 1050 nmol L^−1^ (both EDTA and DFB experiments) were treated with 200 µmol L^−1^ ferrozine (FZ; 5,6-Diphenyl-3-(2-pyridyl)-1,2,4-triazine) at the beginning of experiments. As Fe(II)-FZ_3_ complexes are unavailable for uptake across the plasma membrane [53], a reduction in growth rate suggests a reductive uptake pathway is present in Fe acquisition. FZ concentrations used in our experiments are shown to be suitable in binding ferrous iron without inducing abiotic reduction of Fe(III) species [54]. All Fe-uptake experiments were performed in the dark to prevent the photoreduction of Fe(III) chelates.

### Cellular Fe and P content

All samples and reagents were processed using trace metal clean techniques and acid washed plastic wear (10% HCl v/v, 24 h) in a clean laboratory prior to trace element analysis. SCM1 cells cultured in 300, 550 and 1050 pmol L^−1^ Fe′ were harvested in late exponential phase by centrifugation at 5000 × g for 25 min and then rinsed with Chelex-100 treated SCM media to remove weakly bound surface metals. Cells were digested in acid-cleaned Teflon as follows: refluxing at 100 °C with 16 N quartz distilled (q.d.) HNO_3_ (produced in-house) and H_2_O_2_ (ROMIL UpA™, Cambridge, UK) in a ratio of 3:2 overnight. The liquid was then allowed to evaporate to dryness and 2% q.d. HNO_3_ was added to reflux for an hour to resuspend the dried samples. ^56^Fe and ^31^P concentrations were determined using a Quadrupole ICP-MS Perkin Elmer NexION 350 with Elemental Scientific Flow Injection Auto-sampler (FIAS) in a method optimised for high salt matrixes [34].

### Fe rescue experiments

In order to confirm whether Fe limits SCM1 growth in EDTA-buffered experiments, we performed an “Fe rescue” of experiments, which showed suppressed or no cell growth in the second sub-culture before cultures had become fully acclimated. We supplemented 50 and 150 pmol L^−1^ Fe cultures with additional FeCl_3_ at 120 h resulting in final [Fe′] of 1050 pmol L^−1^. Control cultures received no additional FeCl_3_.

### Data analysis

All statistical analyses were performed using Minitab v.13.1. Data were examined for normality and equal variance prior to student’s *t*-test and analysis of variance (ANOVA) statistical tests. Significant results are reported at the 95% confidence level (*p* < 0.05).

## Results

### Effect of unchelated Fe ion (Fe′) on total nitrite production and specific growth rates

**Fig. 1:**
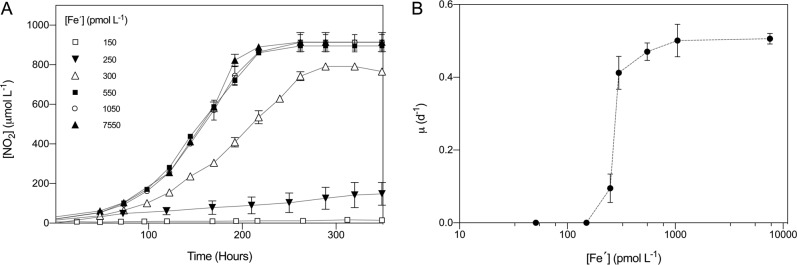
Effect of unchelated inorganic Fe concentration [(Fe´)] on NO_2_^−^ production and specific growth rate (µ) of SCM1. Error bars indicate standard deviation, *n* = 20

Cultures of SCM1 were acclimated to a range of unchelated inorganic Fe concentrations ([Fe′]), as described above. Treatments  with < 250 pmol L^−1^ Fe′ did not support SCM1 growth as evidenced by a total lack of NO_2_^−^ production (Fig. 1a). We observed SCM1 growth in all tested conditions ≥250 pmol L^−1^ Fe′. Treatments at 250 and 300 pmol L^−1^ Fe′ showed suppressed growth curves, producing significantly lower final [NO_2_^−^] (*p* < 0.05), compared with 550–7550 pmol L^−1^ Fe′ cultures, which produced indistinguishable growth curves and maximum [NO_2_^−^] reached.

To provide confirmation that growth inhibition was due to Fe limitation, we supplemented cultures showing little and or no growth in the second experimental sub-culture (where cultures were experiencing Fe stress but before they had become acclimated) with FeCl_3_ to reach a final [Fe′] of 1050 pmol L^−1^ (Supplementary Fig. 3). We observed enhanced NO_2_^−^ production in all supplemented cultures, compared with non-supplemented controls; 150 pmol L^−1^ Fe′ treatments supplemented with additional Fe produced 900 µmol L^−1^ NO_2_^−^ within 11 days and 50 pmol L^−1^ Fe′ cultures produced 900 µmol L^−1^ NO_2_^−^ within 15 days.

Maximum specific growth rates, µ_max_, were observed in 7550 pmol L^−1^ Fe′ treatments (Table 1). The half saturation constant (*K*_*s*_), based on a Monod curve fitted to our data (eq.1) was 361.5 pmol L^−1^ Fe′. Specific growth rate (µ) varied significantly between 250 and 1050 pmol L^−1^ Fe′ treatments (*p* < 0.05), ranging between 27–99% of µ_max_ (Table 1; Fig. 1b). Cultures treated with 250 pmol L^−1^ Fe′ grew at the lowest µ (27% of µ_max_) followed by 300 pmol L^−1^ Fe′ treated cultures which grew at 81% of µ_max_. We observed no significant increase in µ_max_ when [Fe′] > 1050 pmol L^−1^ (*p* > 0.05), suggesting that these cultures were Fe-replete.

**Table 1:** Relationships between Total [Fe], [Fe´], cellular Fe and P concentration, specific growth rate (µ) and relative growth rate (µ/µ_max_). Values in parentheses represent standard deviation

[Fe] (nmol L^−1^)	[Fe´] (pmol L^−1^)	Fe (pmol cell^−1^)	P (pmol cell^−1^)	Fe:P (mmol mol^−1^)	µ (d^−1^)	µ/µ_max_
200	200				0	0
250	250				0.13 (±0.05)	0.27 (±0.02)
300	300	5.0 × 10^−6^ (±4.3 × 10^−6^)	5.5 × 10^−4^ (±1 × 10^−4^)	11.4 (±4.7)	0.41 (±0.05)	0.81 (±0.09)
550	550	6.5 × 10^−6^ (±1.3 × 10^−6^)	3.3 × 10^−4^ (±5.4 × 10^−5^)	18.8 (±3.2)	0.47 (±0.02)	0.93 (±0.05)
1050	1050	1.1 × 10^−4^ (±4.2 × 10^−5^)	2.6 × 10^−3^ (±1.2 × 10^−4^)	39.3 (±11.8)	0.50 (±0.01)	0.99 (±0.09)
7550	7550	0.78 (±0.07)	3.93 (±0.05)	199.95 (±4.9)	0.51 (±0.01)	1 (±0.02)

### Cellular iron quotas

Cellular Fe and P concentrations were measured in response to increasing [Fe′], between 300–7550 pmol L^−1^ Fe′. We observed a scaling between SCM1 cellular Fe and P concentrations, [Fe′] and µ (Table 1). Fe:P varied significantly between treatments growing at different µ (*p* < 0.05), ranging between 11.4 (±4.7) and  199.95 (±4.9) mmol Fe mol^−1^ P. We observed maximum cellular Fe:P in cultures growing at µ_max_.

### Steady state uptake rate of Fe’ and FeDFB

A requirement of steady state uptake rate (*ρ*_ss_) calculations is for cellular Fe quota and specific growth rates to be used when [Fe′] is limiting to growth. We therefore calculated *ρ*_*ss*_ based on the cellular Fe concentration of SCM1 treatments at 300 and 550 pmol L^−1^ Fe′, that were growing at 81 and 93% of µ_max_. Fe′ uptake rate was 1.1 × 10^−22^ mol Fe cell^−1^ h^−1^ ( ± 3.1 × 10^−23^) and the Fe′ uptake rate constant, *K*_in_ (*ρ*_ss_ normalised to [Fe′]) was 4.5 × 10^−13^ L cell^−1^ h^−1^ (±1.2 × 10^−13^). The uptake rate of FeDFB was significantly lower than Fe′ (*p* < 0.05) at 2.8 × 10^−21^ L cell^−1^ h^−1^ (±1.6 × 10^−22^) equating to an uptake rate constant of 2.8 × 10^−15^ L cell^−1^ h^−1^ (±1.6 × 10^−16^).

### SCM1 siderophore production and FeDFB uptake

**Fig. 2:**
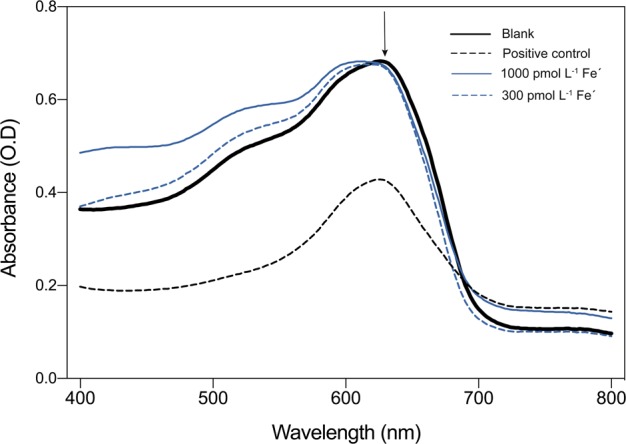
Absorbance spectra of SCM1 culture supernatant with 300 (blue dashed line) and 1050 pmol L^−1^ Fe (blue solid line) measured between 400–800 nm following treatment with Chrome Azurol S (CAS) assay solution. Siderophore production was denoted as a decrease in absorbance from the blank (uninoculated SCM medium—black solid line) at 630 nm, marked with an arrow. 10 pmol L^−1^ DFB solution was used as a positive control (see methods for further details)

**Fig. 3:**
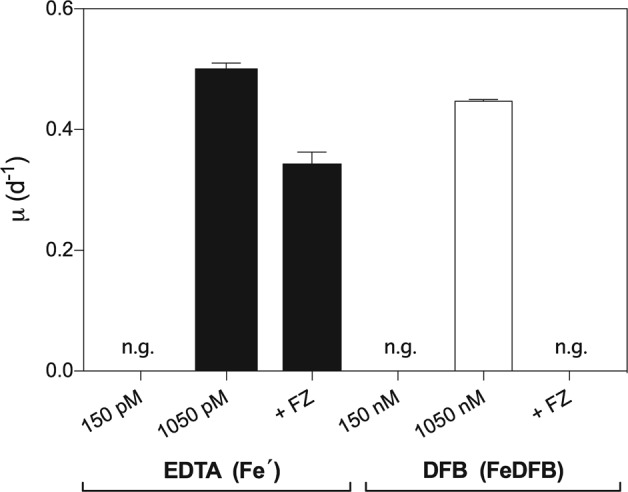
Effect of Fe on SCM1 specific growth rate (µ) in EDTA-buffered experiments wherein the available substrate is Fe´ (solid bars) and DFB buffered experiments wherein there is negligible Fe´ and all Fe is bound to DFB (FeDFB—open bars), and effect of Fe(II) specific chelator, ferrozine (FZ) on specific growth rates of SCM1 in these cultures (+FZ). n.g. indicates no SCM1 growth. Concentrations on *x*-axis indicate the available substrate (i.e. Fe´ or FeDFB). Error bars indicate standard deviation, *n* = 11

We conducted a CAS assay to determine whether SCM1 produces siderophores. In this assay, there was no evidence for siderophore production in the medium of either 300 pmol L^−1^ or 1050 pmol L^−1^ Fe′ treatments, as the absorbance spectra of culture supernatants did not differ significantly from the blank (Fig. 2). However, this result does not completely rule out the production of very low concentrations (<10 pmol L^−1^ detection limit) of siderophores by SCM1. The ability of SCM1 to grow using siderophore bound Fe was examined using growth media wherein all Fe was bound to the hydroxamate siderophore, DFB (FeDFB), which reduces [Fe′] to negligible concentrations (see Methods). We observed no SCM1 growth at 150 nmol L^−1^ FeDFB. Only treatments with 1050 nmol L^−1^ FeDFB displayed SCM1 growth, reaching µ of 0.44 d^−1^ (Fig. 3); however, this is significantly lower than with EDTA-buffered treatments with 1050 pmol L^−1^ Fe′ (*p* < 0.05), which reached µ of 0.50 d^−1^.

### The role of reduction in Fe uptake

In order to determine whether reduction of Fe(III) to Fe(II) is a component of SCM1 Fe acquisition, we examined the effect of ferrozine (FZ) addition on SCM1 growth rate. FZ significantly reduced µ in both EDTA and DFB buffered cultures (*p* < 0.05), compared with the untreated controls (Fig. 3) suggesting a requirement for Fe(III) reduction in Fe uptake. In EDTA treatments, addition of FZ reduced µ by 30%, compared to DFB treatments whereby FZ reduced µ by 100%.

## Discussion

### SCM1 has an elevated K_s_ compared with marine phytoplankton driven by a reduced affinity for Fe’

**Fig. 4:**
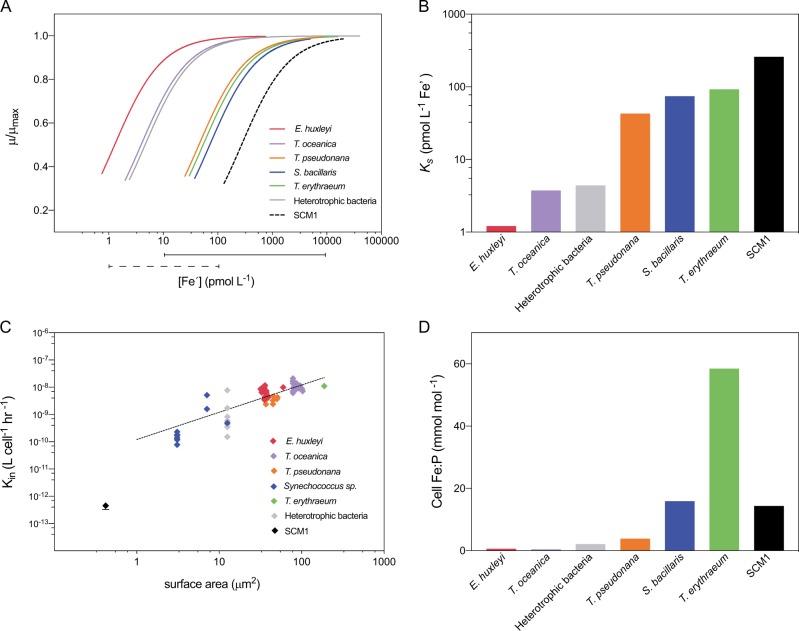
**a** Monod curves fitted to compiled phytoplankton and heterotrophic bacteria data showing the effect of [Fe´] on relative growth rate (µ/µ_max_) compared with data from this study (SCM1). The range of oceanic total [Fe] (solid line) and [Fe´] (dashed line) from refs. [85–92] are displayed below the plot. **b** Half saturation constants (*K*_s_) based on Monod curves fitted to compiled data. **c** Fe´ uptake rate (L cell^−1^ h^−1^) of phytoplankton, heterotrophic bacteria and SCM1 as a function of surface area. **d** Critical cellular Fe:P as estimated from linear regression of relative growth rate (in Fe limited cells) against cellular Fe:P (see Supplementary Methods for more detail). Published datasets and references used can be found in Supplementary Tables 1 and 2 [93, 94]

In order to place our results into the context of the microbial community, we compared the Fe-requirements of SCM1 with published datasets of a range of environmentally important marine microorganisms (Fig. 4). There is a great wealth of data surrounding the role of Fe on marine phytoplankton growth, motivated by the finding that iron limits primary productivity in 30% of the world’s surface ocean [55, 56]. We compared the effect of [Fe′] on growth rate (including the half saturation constant, *K*_s_), the Fe′ uptake rate and critical cellular Fe:P quota (Supplementary Methods) between published phytoplankton, marine heterotrophic bacterial datasets and SCM1 data from this study.

We observed that SCM1 displays the greatest half saturation constant (*K*_s_) for Fe′ amongst the organisms assessed, despite not showing the highest critical cellular Fe quota (Fig. 4). In phytoplankton and marine bacteria, the critical cellular Fe quota (i.e. the minimal cellular Fe demand to reach µ_max_—Supplementary Fig. 4) is an important determinant in *K*_s_ as surface area normalised Fe′ uptake rate is remarkably consistent [30]. The similarity in rate constants across a range of taxonomically diverse phytoplankton and marine bacteria reflects the selective pressure placed on phytoplankton over time to evolve efficient Fe-uptake systems in environments wherein Fe is chronically low [57]. This is evident in the genomes of phytoplankton and heterotrophic bacteria, which commonly encode for Fe-specific membrane transport proteins (e.g. FeoA/B/C, AfuA, FTR1) in addition to general metal cation transporters [58, 59]. However, SCM1 does not follow the observed proportionality in uptake rate constants that have emerged from phytoplankton and bacterial data, displaying a reduced affinity for Fe′ relative to cell surface area (Fig. 4c; *p* < 0.05). Perhaps unsurprisingly, genes encoding for Fe-specific membrane transport proteins identified in eukaryotes and prokaryotes are absent from the genome of SCM1. Instead it appears that SCM1 only has genes encoding for generalist divalent cation transporters belonging to the Tro and ZIP ABC transporter families (Supplementary Table 3; Nmar_0328, Nmar_0330, Nmar_0331, Nmar_0329, Nmar_1130 and Nmar_1662). We therefore speculate that the high *K*_s_ of SCM1 is driven by a reduced affinity for Fe′ due to a lack of Fe-specific metal uptake proteins and not by a distinctly elevated critical cellular Fe quota. The only marine AOA documented to have genes for Fe-specific membrane uptake proteins is *Candidatus* Nitrosopelagicus brevis str. CN25 (herein *Ca*. N. brevis) (Supplementary Table 3), encoded by T478_0963. However, an initial search of the TARA metagenomic database (Supplementary Table 4 and Supplementary Fig. 5), returns a greater number of *Thaumarcheotal* hits for genes homologous to non-specific metal transporters homologous over *Ca.* N. brevis-related Fe^2+^ specific transporters (FeoB encoded by gene T478_0963), suggesting that SCM1 Fe-uptake dynamics is a representative of Fe-uptake processes in marine AOA.

### Fe requirement of SCM1

Our data compilation indicates that *Synechococcus bacillaris* and *Trichodesmium erythraeum*, have the greatest critical cellular Fe:P required to reach µ_max_ (Fig. 4 and Supplementary Fig. 4). This is unsurprising in both cases: the N_2_-fixation capacity of *T. erythraeum* drives a high Fe-requirement due to the Fe-rich nitrogenase proteins [60–62]; the elevated Fe:P in *S. bacillaris* compared with eukaryotic diatoms and haptophytes can be explained by a higher proportion of Fe-rich photosystem I versus photosystem II [63, 64]. Given that SCM1 does not have photosynthetic or nitrogen fixation capabilities, our observed Fe quota may be greater than initially expected. However, proteomic analyses indicate that ferredoxin, an Fe-S protein encoded by the gene Nmar_0239, is the most highly expressed protein in exponentially growing SCM1 cells [65]. Analyses of *Ca*. N. brevis also revealed that Fe-S proteins were among the most highly expressed genes and proteins [66, 67]. In both cases, Fe-S proteins are co-expressed with key ammonia-monoxygenase modules implying that Fe-S proteins play a central role in the electron transport chain of *Thaumarchaeota* in addition to Cu as previously suggested [22, 23]. It is yet to be explored whether the presence of cytochromes in AOB, drives a similar, lower or greater Fe-requirement in AOB in doing so influencing the niche differentiation between AOB and AOA in the oceans, in addition to ammonia affinity [25, 26].

### Utilisation of siderophore bound Fe through a reductive uptake pathway

In the oceanic surface waters, >99.9% of Fe is complexed by organic ligands [25], thereby rendering the concentration of unchelated inorganic Fe (Fe′) to vanishingly low concentrations and creating a requirement for photochemical or redox mechanisms to maintain an adequate supply of Fe′ [68]. The high [Fe′] required to see growth in our SCM1 cultures (>150 pmol L^−1^ Fe′), strongly suggests that SCM1 is able to access another source of Fe in the oceans to fulfil their Fe requirements, or else AOA would unlikely be so widespread in the marine environment [10]. We observed that while SCM1 is not able to produce siderophores to enhance access to Fe under low [Fe′] (Fig. 2), SCM1 can grow using Fe bound to the strong hydroxamate siderophore, DFB, likely through a reductive uptake pathway as demonstrated in range of marine phytoplankton (Fig. 3) [28, 54] or an alternative yet unknown uptake pathway. The use of a reductive Fe-uptake pathway (for both EDTA and DFB) agrees with bioinformatic evidence showing that SCM1 only has metal uptake proteins capable of divalent cation transport across the inner membranes (Supplementary Table 3), necessitating the reduction of ferric Fe-complexes before they can be imported into the cell. As our experiments were performed in the dark, we can exclude the possibility of abiotic photoreduction of organic chelates as a source of Fe(II) to either divalent uptake pathways and can be confident that SCM1 is actively reducing the Fe-organic complex.

Ferric reductases present in phytoplankton have been characterised [28, 54]; however, to date only one archaeal ferric reductase, from *Archaeoglobus fulgidus*, has been isolated and characterized [69]. The ferric reductase responsible for reducing ferric-chelates before uptake across the inner membrane has not yet been identified in SCM1. To identify candidate ferric-reductase proteins, we performed a BLASTp search of all known ferric-chelate reductases (prokaryotic and eukaryotic) against the SCM1 genome (see Supplementary Methods and Supplementary Table 5). Our search highlighted one candidate gene, Nmar_0180, which returned a significant e-value (e-value threshold defined as e^−25^) and high bit score. Nmar_0180 encodes a NADP oxidoreductase F420-dependent coenzyme (Supplementary Table 6), which shows significant sequence homology with proteins that have ferric-chelate reductase (NADPH) activity (GO: 0052851) identified from two archaea, *Halobacterium salinarum* and *Methanocaldococcus jannaschii*. Further work on isolating and characterizing the activity of protein encoded by Nmar_0180 is required in order to ascertain whether this protein is responsible for reducing Fe(III) before uptake by divalent metal transporters.

#### Surface water nitrification

Several studies have attributed the low rates of nitrification rates in surface waters to competition with marine phytoplankton for ammonium [16, 17, 25, 26] suggesting that it is only when phytoplankton become light limited below the photic zone that this competition is removed. Although this is generally supported by coupled depth profiles of organic matter remineralisation (which liberates NH_4_^+^) and nitrification rates [70–72], rates of nitrification are significantly variable in surface waters [73] and do not always relate to ammonium availability and AOA abundance. For example, Smith and colleagues (2015) showed that even when there is ample substrate and low phytoplankton abundance, only a small fraction of the NH_4_^+^ pool is oxidised, suggesting that an additional factor controls nitrification in surface waters. The reduced affinity of SCM1 for both DFB and unchelated Fe compared with marine phytoplankton and heterotrophic bacteria (Fig. 4) suggests that AOA are also uncompetitive for Fe in the photic zone providing a plausible additional factor which limits AOA growth in surface waters. Enhanced nitrification rates have been observed in oceanic surface waters during natural  Fe fertilisation events in the Southern Ocean [74, 75] whether this is driven by an increased supply of  Fe remains an interesting avenue to be explored in light of our experimental results.

Although our siderophore-uptake experiments were only performed with DFB, we hypothesise that the potential reductive uptake strategy of SCM1 may confer SCM1 with the ability to access other forms of chelated Fe. Iron reductases identified in other marine microorganisms (both prokaryotes and eukaryotes) have been shown to be ‘generalist’, being able to reduce a wide range of Fe-chelates [30, 54]. As SCM1 is able to reduce FeDFB, which is a hexadentate siderophore forming a very stable octahedral coordination, it is plausible that SCM1 is also able to reduce weaker bound Fe-chelates with a greater reducing potential, such as exopolysaccharides and humic acids [30]. However, this remains to be experimentally explored as other factors, such as the charge of the ligands are important determinants in the ability of ferric reductase to reduce Fe-chelates [28]. The nature of the Fe-reductase that SCM1 utilises may also clarify why SCM1 has a lower affinity for FeDFB compared to other marine microorganisms (Supplementary Fig. 6), ultimately shedding light on the fundamental driving factors behind the reduced competitiveness of SCM1 in the photic zone.

#### Below the photic zone

Despite several observations of nitrification in surface waters [73], maximum rates of nitrification and abundances of AOA are repeatedly observed at the base of the photic zone [10, 70, 76]. As described previously, this is often attributed to an enhanced supply of NH_4_^+^ in association with remineralising organic matter [16, 71] and the alleviation of phytoplankton competition. However, additions of the trace metal chelator TETA in the equatorial Pacific caused a reduction in nitrification rates by up to 23% between 150–300 m depth [71], implicating a key role for trace metals in shaping the distribution of AOA and nitrification rates below the photic zone. As phytoplankton become light limited with increasing depth, AOA competition with phytoplankton for Fe would be alleviated in a similar way that has been suggested for NH_4_^+^ [16, 17]. Marine heterotrophic bacteria also decline in abundance at the base of the photic zone [10, 77, 78] likely driven by the reduction in dissolved organic carbon (DOC) at this depth which numerous studies have shown to be limiting to heterotrophic bacterial growth [79–82]. Iron additions alone have not been shown to enhance heterotrophic bacterial growth [83, 84] or have only been shown to enhance growth through an indirect effect by the enhanced phytoplankton DOC production [82–86], suggesting that it is unlikely that heterotrophic bacteria compete with AOA for Fe at the base of the photic zone. Both a reduction in the Fe consumption from the phytoplankton and marine bacterial communities would enable AOA relatively un-contested access to Fe at the base of the photic zone. We speculate that such a niche may provide AOA with sufficient Fe to reach their metabolic demands despite possessing relatively low affinity Fe-uptake pathways. Moreover, the reduced competition for Fe comes with a reduced evolutionary pressure to improve Fe-uptake pathways and so goes some way to explaining why SCM1 appears to be a poor competitor for Fe.

## Conclusion

Our work is the first to examine the role of Fe in *Thaumarchaeota* growth, providing evidence that the marine ammonia-oxidising archaeon *Nitrosopumilus maritimus* SCM1 has the highest half saturation constant for Fe′ of any microorganism examined to date. We showed that whilst unable to synthesise siderophores, SCM1 is able to access Fe bound to the organic siderophore DFB, and likely employs a reductive uptake pathway as part of it’s Fe acquisition strategy.

Our work presents an additional explanation as to why nitrification rates are often lowest in surface waters and greatest rates are observed at the base of the photic zone [16, 70, 71]. This provides greater depth to our current understanding of AOA distribution which currently focuses largely on NH_4_^+^ availability [16]. We suggest that AOA are potentially outcompeted for Fe in surface waters by phytoplankton and heterotrophic bacteria, which have a greater affinity for important Fe substrates, supporting findings of previous work indicating that other factors besides NH_4_^+^ availability regulate nitrification rates in surface waters [11, 13, 17]. We suggest that at the base of the photic zone, where phytoplankton and heterotrophic bacteria are limited by other factors (light and DOC), AOA are able to proliferate leading to the highest observed rates of nitrification. The curious lack of specificity in AOA Fe-uptake systems in contrast with phytoplankton and marine bacteria [58, 59] also agrees with the growth of AOA in an environment wherein there is reduced competition for Fe, such as the base of the photic zone.

Future research should focus on examining Fe-uptake dynamics in additional species of marine AOA, to develop a more encompassing view of marine AOA ecophysiology, which is not purely based on the cultivation results of one marine AOA. Additionally, future work should identify the responsible ferric reductase for archaeal Fe uptake and determine the proteins driving the Fe demand of SCM1 at the genomic and proteomic level. Furthermore, our laboratory experiments should be corroborated with ship-board incubation experiments to determine whether AOA are Fe limited, starting in regions of persistent Fe scarcity, such as the Southern Ocean.

## Supplementary information

Supplementary Materials

Supplementary Table 1

Supplementary Table 2

Supplementary Table 3

Supplementary Table 4

Supplementary Table 5

Supplementary Table 6
