# Supplementary material for: Iron requirements and uptake strategies of the globally abundant marine ammonia-oxidising archaeon, Nitrosopumilus maritimus SCM1
Source: ISME J. 2019 May 10;13(9):2295–305. doi: 10.1038/s41396-019-0434-8 (PMC6776035; doi:10.1038/s41396-019-0434-8)
Supplement: Supplementary file 1 — Supplementary Materials [file 41396_2019_434_MOESM1_ESM.docx]

# Supplementary Material

# Iron requirements and uptake strategies of the globally abundant marine ammonia-oxidising archaeon, *Nitrosopumilus maritimus* SCM1

Roxana T. Shafiee*^1^, Joseph.T. Snow^1^, Qiong Zhang^1^, Rosalind E.M. Rickaby^1^

^1^ Department of Earth Sciences, South Parks Road, University of Oxford, Oxfordshire, OX1 3AN, United Kingdom.

* Corresponding author

roxana.shafiee@earth.ox.ac.uk

# Supplementary Methods

### **Calculation of the critical cellular Fe:P required to reach µ_max_**

In comparing iron quotas of microorganisms, it is necessary to distinguish between intracellular iron incorporated into functional proteins, needed to support growth, versus iron quotas representing iron storage and ‘luxury uptake’. In our comparison, we were interested in intracellular iron associated with supporting growth, as this is what is relevant in the context of Fe´ dose response curves. A first-order linear relationship in iron-limited diatoms between [Fe´], Fe:C and growth rate (µ) whereby the x-axis intercept (at the point where the linear regression crosses µ_max_ – Supplementary Figure 2) represents the minimum Fe:C required to achieve µ_max_ (herein referred to as critical cell Fe:P) has been demonstrated (1). Assuming that the same applies for other marine microorganisms, we plotted relative growth rate against cell Fe:P in Fe-limited cells and fitted data with a linear regression. The critical cell Fe:P was defined as the x-intercept when y (µ_max_) is equal to one, calculated using the linear regression equation. Original data used in our comparison (1-7) is available in Supplementary Table 1. Original Fe:C data was converted to Fe:P data for comparison assuming a “Redfield” C:N:P stoichiometry of 106:16:1.

### **Search for candidate ferric reductases**

To date, no genes in the genome of SCM1 have been annotated with ferric reductase functionality. In order to identify potential ferric reductases, we compiled a database of known ferric reductases from archaea, eukaryotes, and bacteria also including proteins which have been annotated as having ferric-chelate reductase (NADPH) activity in the gene orthology (GO) database (Supplementary Table 4). We performed a BLASTp ferric reductase proteins against the SCM1 genome to identify any candidate ferric reductases using an E-value threshold of 1 x 10^-25^ (Supplementary Table 5).

**Flow cytometry**

In order to corroborate the relationship between cell counts and [NO_2_] under Fe-deplete conditions, we measured cell densities from 250 pmol L^-1^ Fe**´** cultures at different points in the SCM1 growth curve (equating to a range of [NO_2_] from 9 – 750 µmol L^-1^. Samples were stained with SYBR Green I (final concentration = 10^-4^ dilution from a concentrated stock of SYBR Green, Thermo Fisher Scientific) prior to imaging with Amnis Imagestream MKII imaging flow cytometer housed in the Dunn School of Pathology, University of Oxford. We found that our data agrees well with previous published data under Fe-replete conditions (15), showing that the changes in nitrite at low concentration are driven by differences in cell density rather than just a constant number of cells actively oxidising ammonia in culture (Supplementary Fig. 4).

**Scanning electron microscopy**

Fe-limited (250 pmol L^-1^) and Fe-replete (1000 pmol L^-1^) cell cultures were harvested by centrifugation into a concentrated cell suspensions. Cell suspensions were applied to poly-L-lysine coated glass coverslips and were then fixed with 2.5% glutaraldehyde and allowed to adhere for 5 min. Glass coverslips were rinsed 3x with 0.1M phosphate buffer (pH 7.2) and then underwent secondary fixation with 1% OsO_4_ for 45 min. Cells on coverslips were rinsed with water, dehydrated through a graded ethanol series to absolute ethanol, and then dried chemically with HDMS. Coverslips were mounted on carbon adhesive tape and splutter coated with 10 nm Au. Cells were examined using a GeminiSEM at an accelerating voltage of 3 kV and magnifications of × 5,000 – 100,000. Cell dimensions used for calculation of cell surface area in uptake rates were used from Fe-limited treatments (Supplementary Fig. 5B) as it is a requirement that steady state uptake rate is calculated under non-Fe saturating conditions.

**Search of TARA metagenomic database**

*Candidatus* Nitrosopelagicus brevis (herein refered to as *Ca* N. brevis) is the only ammonia-oxidising archaeon we examined which possesses a ferrous iron transporter (encoded by *T478_0963*). We explored whether Fe-specific transporters play a more significant role in marine *Thaumarcheotal* Fe uptake in the open ocean, compared with non-specific metal transporters used by SCM1 and related AOA (Supplementary Table 3). Using the Ocean Gene Atlas search facility of the TARA oceans metagenomic database, we examined whether homologs of *Ca* N. brevis Fe^2+^ uptake gene (*T478_0963*) and its homologs were present in greater abundances compared with the non-specific metal transporters used by *N. maritimus*. To investigate this, we searched the TARA oceans database using the ocean gene atlas (OGA - http://tara-oceans.mio.osupytheas.fr/ocean-gene-atlas/) for homologs of *N. maritimus* unspecific metal uptake transporter genes (*Nmar_0328*, *Nmar_0330*, *Nmar_0331*, *Nmar_0329*, *Nmar_1130*, *Nmar_1662*) and *Ca* N. brevis *FeoB* transporters (*T478_0963*) using FASTA sequences downloaded from UniProt. This search returned any homologous genes over the threshold E-value of E^-10^ and also ascribes taxonomic classification to hits, usually down to the order level. Our aim was to compare only hits belonging to *Thaumarchaeota*, as these represent the marine AOA community.

The analysis returned no hits of homologs of *N. brevis* T478_0963 ascribed to organisms belonging to the *Thaumarcheota* phylum indicating that the Fe-specific uptake proteins and their homologs of *N. brevis* are below the limit of detection in metagenomic studies across all TARA stations. The only archaeal homologs returned from the search were ascribed to the order *halobacteria* within the phylum *Euryarchaeota* **(Supplementary Figure 6)**. The analysis however returned multiple *Thaumarcheota* hits for homologs of all *N. maritimus* unspecific metal uptake genes (nmar_0328, nmar_0330, nmar_0331, nmar_0329, nmar_1130, nmar_1662): of these, 68-94% of all archaeal hits were ascribed to the *Nitrosopumilales* order (Supplementary Figure 6) These results suggest that genes relating to *N. maritimus* metal transport systems are vastly more prevalent in the wider oceanic environment than those relating to the high-affinity *N. brevis* *FeoB* – which did not return any hits.

# Supplementary Figures

**Supplementary Figure 1.** Correlation between cell counts and nitrite concentrations in 250 pmol L^-1^ experiments (red squares) compared with data from Amin et al (2013) at replete Fe conditions (7500 pmol L^-1^ Fe´), Martens-Habbena *et al* (2009) and Könneke *et al* (2005)


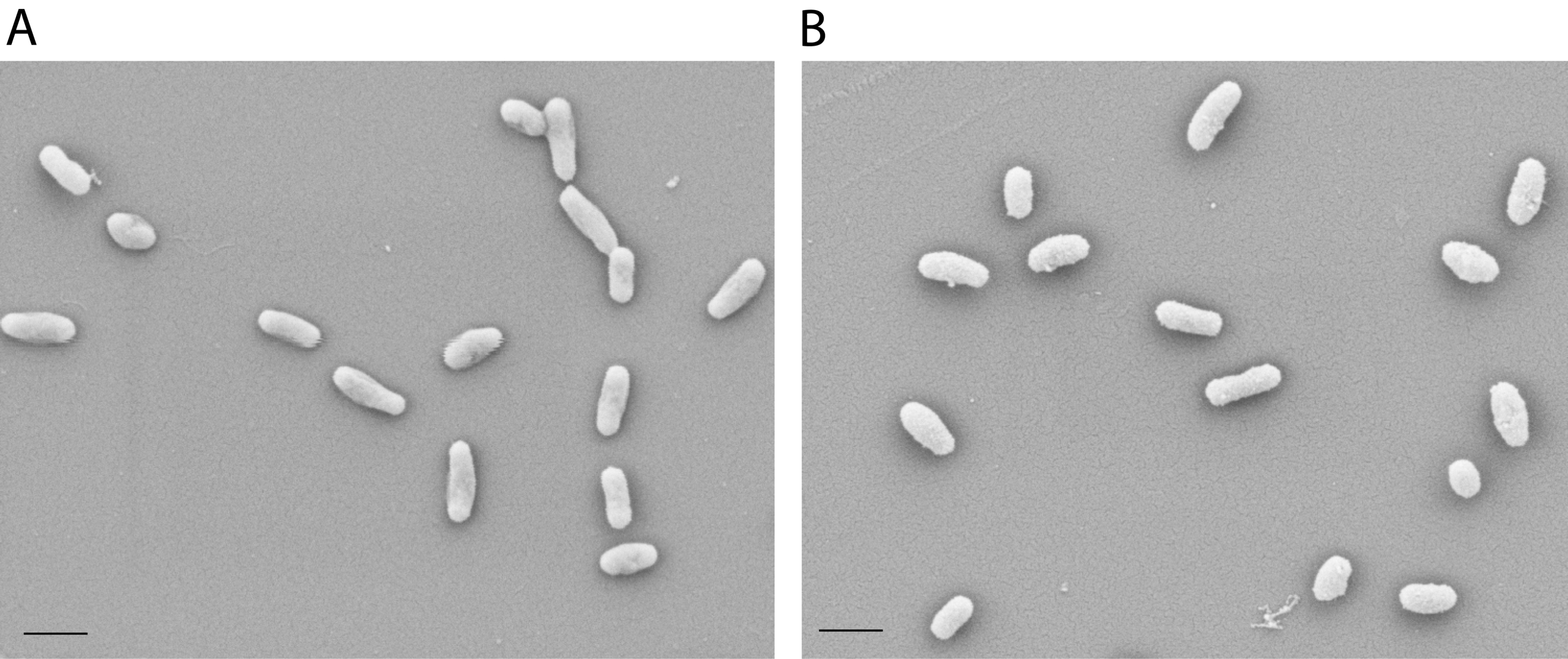


**Supplementary Figure 2.** Scanning electron micrograph of A) SCM1 cells grown under Fe-replete (1000 pmol L^-1^ Fe´) conditions and B) Fe-deplete (250 pmol L^-1^ Fe´) conditions. Scale bar in both images represents 0.5 µm.


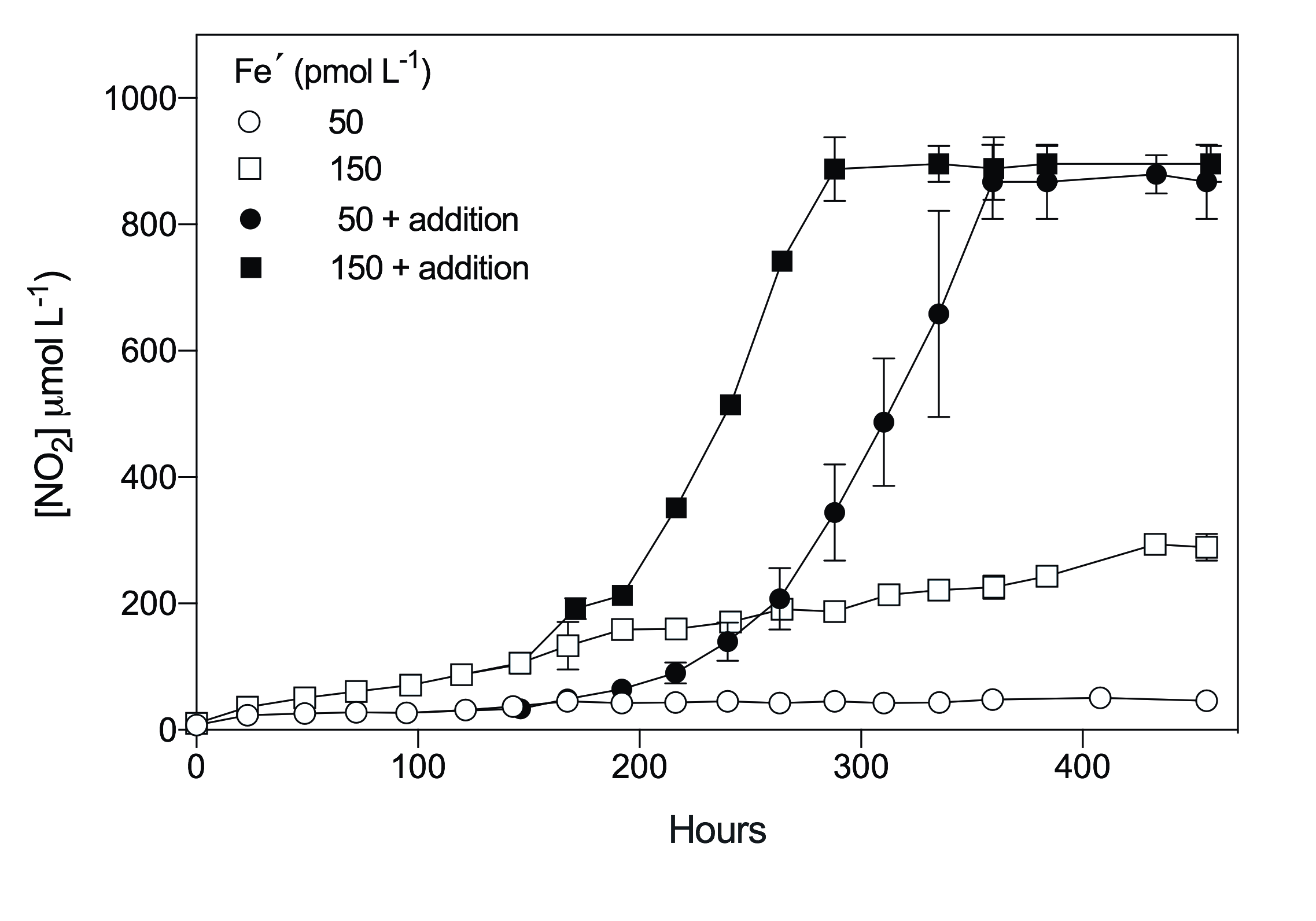


## Supplementary Figure 3. The effect of addition of Fe at 120 hours to cultures of SCM1 treated with in experiments of 50 pmol L^-1^ and 150 pmol L^-1^ [Fe´] prior to full acclimation to [Fe´]. Final [Fe´] in amended cultures was 1000 pmol L^-1^. Control cultures received no additional Fe. Error bars indicate standard deviation, n = 6.

## Supplementary Figure 4 . The linear relationship between specific growth rate (µ) and cellular iron quota (Fe:P) in iron limited cells of phytoplankton and SCM1 (i.e. below relative growth rate = 1). X-axis intercept of the point at which linear regression lines crossed µ_max_ (y=1) were used as the Fe:P ratio indicative to minimal Fe requirement to achieve maximum growth rates.

**Supplementary Figure 5.** Percentage of archaeal hits ascribed to orders *Nitrosopumililales, Nitrososphaeria, Halobacteria, Methanomicrobia* returned from TARA oceans database search of *Nitrosopumilus maritimus* metal uptake genes (*Nmar_0328, Nmar_0330, Nmar_0331, Nmar_0329, Nmar_1130, Nmar_1662*) and *Ca.* N brevis *T478_0963*.

**Supplementary Figure 6.** FeDFB uptake rate (L cell^-1^ hr^-1^) of phytoplankton (refs.10-14) and SCM1 as a function of cell surface area.

**Supplementary References**

1. Sunda WG, Huntsman SA. Iron uptake and growth limitation in oceanic and coastal phytoplankton. Marine Chemistry. 1995 Aug 1;50:189-206.
2. Schulz KG, Zondervan I, Gerringa LJ a, et al. Effect of trace metal availability on coccolithophorid calcification. Nature. 2004;430:673–6. ﻿
3. Sunda WG, Huntsman S. Interrelated influence of iron, light and cell size on marine phytoplankton growth. Nature. 1997;390:389–92. ﻿
4. Sunda W, Huntsman S. High iron requirement for growth, photosynthesis, and low-light acclimation in the coastal cyanobacterium *Synechococcus bacillaris*. Front Microbiol. 2015;6:1–13. ﻿
5. Berman-Frank I, Cullen JT, Shaked Y, Sherrell RM, Falkowski PG. Iron availability, cellular iron quotas, and nitrogen fixation in *Trichodesmium*. Limnol Oceanogr. 2001;46:1249–60. ﻿
6. Shi D, Kranz SA, Kim J-M, Morel FMM. Ocean acidification slows nitrogen fixation and growth in the dominant diazotroph *Trichodesmium* under low-iron conditions. Proc Natl Acad Sci .2012;109:E3094–100.
7. Boatman TG, Oxborough K, Gledhill M, Lawson T, Geider RJ. An integrated response of *Trichodesmium erythraeum* IMS101 growth and photo-physiology to iron, CO2, and light intensity. Frontiers in microbiology. 2018;10:624.
8. Santoro AE, Dupont CL, Richter RA, Craig MT, Carini P, McIlvin MR, et al. Genomic and proteomic characterization of “*Candidatus* Nitrosopelagicus brevis” an ammonia-oxidizing archaeon from the open ocean. Proc Natl Acad Sci U S A. 2015;112:1173–8
9. Valasatava Y, Rosato A, Banci L, Andreini C. MetalPredator: a web server to predict iron–sulfur cluster binding proteomes. Bioinformatics. 2016;32:2850-2.
10. Strzepek RF, Maldonado MT, Hunter KA, Frew RD, Boyd PW. Adaptive strategies by Southern Ocean phytoplankton to lessen iron limitation: Uptake of organically complexed iron and reduced cellular iron requirements. Limnol Oceanogr. 2011;56:1983–2002.
11. Maldonado MT, Price NM. Reduction and transport of organically bound iron by Thalassiosira oceanica (Bacillariophyceae). J Phycol. 2001;37:298–309.
12. Shaked Y, Kustka a B, Morel FMM, Erel Y. Simultaneous determination of iron reduction and uptake by phytoplankton. Limnol Oceanogr. 2004;2:137–45.
13. Lis H, Shaked Y, Kranzler C, Keren N, Morel FMM. Iron bioavailability to phytoplankton: an empirical approach. ISME J. 2014;9(4):1003–13.
14. Hassler CS, Schoemann V. Bioavailability of organically bound Fe to model phytoplankton of the Southern Ocean. Biogeosciences. 2009; 6: 2281-2296.
15. Amin SA, Moffett JW, Martens-Habbena W, Jacquot JE, Han Y, Devol A, et al. Copper requirements of the ammonia-oxidizing archaeon *Nitrosopumilus maritimus* SCM1 and implications for nitrification in the marine environment. Limnol Oceanogr. 2013;58:2037–45
